# CIAPIN1 Targeted NHE1 and ERK1/2 to Suppress NSCLC Cells' Metastasis and Predicted Good Prognosis in NSCLC Patients Receiving Pulmonectomy

**DOI:** 10.1155/2019/1970818

**Published:** 2019-04-09

**Authors:** Jian Wang, Ying Zhou, Li Ma, Shannan Cao, Wei Gao, Qingqing Xiong, Kaiyuan Wang, Lili Yang

**Affiliations:** ^1^Department of Immunology, Tianjin Medical University Cancer Institute and Hospital; National Clinical Research Center of Cancer; Key Laboratory of Cancer Prevention and Therapy; Tianjin's Clinical Research Center for Cancer; Key Laboratory of Cancer Immunology and Biotherapy, Tianjin 300060, China; ^2^Department of Neuro-Oncology and Neurosurgery, Tianjin Medical University Cancer Institute and Hospital, Tianjin 300060, China; ^3^Clinical Laboratory, Yantai Affiliated Hospital of Binzhou Medical University, Yantai 264000, China; ^4^Key Lab for Immunology in Universities of Shandong Province, School of Clinical Medicine, Weifang Medical University, Weifang 261053, China; ^5^Department of Hepatobiliary Surgery, Tianjin Medical University Cancer Institute and Hospital; National Clinical Research Center for Cancer; Key Laboratory of Cancer Prevention and Therapy, Tianjin 300060, China; ^6^Department of Anesthesiology, Tianjin Medical University Cancer Institute and Hospital, Tianjin 300060, China

## Abstract

**Objective:**

Cytokine-induced apoptosis inhibitor 1 (CIAPIN1) acts as a downstream effector of the receptor tyrosine kinase-Ras signaling pathway and has been reported as a candidate tumor suppressor gene in various cancers. Our current study was aimed at investigating the prognostic impact of CIAPIN1 on Non-Small-Cell Lung Carcinoma (NSCLC) patients and the effect of CIAPIN1 on NSCLC A549 cells' metastasis.

**Methods:**

Western blot analysis was applied to detect CIAPIN1 expression; Kaplan-Meier survival analysis was used to evaluate the effect of CIAPIN1 on NSCLC patients' prognosis. Wound healing assay, Transwell chamber invasion analysis, and tumorigenicity assay in BALB/c nude mice were used to measure the metastasis potential of A549 cells.

**Results:**

We found that CIAPIN1 overexpression indicated good survival duration during the follow-up period. CIAPIN1 overexpression inhibited the migration, invasion, MMPs, and EMT-associated markers in A549 cells. Further, NHE1 (Na+/H+ exchanger 1) expression and ERK1/2 phosphorylation decreased along with CIAPIN1 upregulation. Importantly, treating A549 cells with CIAPIN1 overexpression with the NHE1-specific inhibitor, Cariporide, further inhibited the metastatic capacity, MMP expression, EMT-associated markers, and phosphorylated ERK1/2. Treatment with the MEK1-specific inhibitor, PD98059, induced nearly the same suppression of CIAPIN1 overexpression-dependent metastatic capacity, MMP expression, and EMT-associated markers as was observed with Cariporide. Further, Cariporide and PD98059 exert synergistical suppression of A549 cells' metastatic capacity.

**Conclusion:**

Thus, the current results implied a potential management by which CIAPIN1 upregulation may have a crucial effect on the suppression of NSCLC, indicating that overexpression of CIAPIN1 might serve as a combination with chemotherapeutical agents in NSCLC therapy.

## 1. Introduction

Lung cancer has been considered one of the leading causes of cancer-related mortality owing to late diagnosis and limited treatment intervention in the world with one million new cases annually in terms of incidence and mortality [1–4]. Lung cancer mainly consists of small-cell lung cancer (SCLC) and non-SCLC (NSCLC) [5]. Patients diagnosed with NSCLC (squamous cell carcinoma, adenosquamous cell carcinoma, and large-cell carcinoma) account for almost 80% of lung cancer patients [6]. Although the working molecular mechanisms underlying lung cancer progress have obviously developed along with advanced molecular biology techniques, the 5-year survival rate of lung cancer is 15%, which showed no significant improvement compared with 13% [7, 8]. Additionally, the management of patients with NSCLC is based on systemic chemotherapy, and though chemotherapy could prolong survival among patients with advanced disease, clinically significant adverse effects reduce its usefulness since excessive toxicity is often reported [9]. A major challenge against lung cancer has been thought to seek novel therapeutic targets that may complement current chemotherapy regimens [10].

Cytokine-induced apoptosis inhibitor 1 (CIAPIN1) originally named as anamorsin or V62 is a newly identified apoptosis-associated protein. A series of reports demonstrated that CIAPIN1 shows no homology with Bcl-2, caspase, IAP families, or other signal transduction molecules and has been proved to participate in regulating the RAS signaling pathway [11–14]. CIAPIN1 was also proved to exert a pivotal effect on some malignant cancers such as gastric cancer, hepatocellular carcinoma, and renal cancer [14, 15]. Furthermore, CIAPIN1 was found to be ubiquitously distributed in both normal fetal and tumor tissues, with high expression in actively metabolic tissues [16, 17]. Thus, CIAPIN1 might be likely involved in important physiological functions in cancer.

The human NSCLC cell line A549 was first developed by Giard et al. in 1972 [18], which can be cultured *in vitro* easily and is widely used as an *in vitro* model for drug metabolism and function assessment [19]. In our study, we tried to investigate the correlation of CIAPIN1 and lung cancer patients' prognosis, as well as the role of CIAPIN1 in A549 cells' migration and invasion.

## 2. Experimental Procedures

### 2.1. Patients and Collection of Samples

This study was performed according to the recommendations of the biomedical research guidelines involving human participants constructed by the National Health and Family Planning Commission of China. The protocols used in the study were approved by the Ethical Committee of Tianjin Medical University Cancer Institute and Hospital. Written informed consent to use excess pathological specimens for research was obtained from each participator in accordance with the Declaration of Helsinki. Collectively, a total of 106 NSCLC patients receiving complete pulmonary resection and systematic lymph node dissection were enrolled from the Lung Carcinoma Department of Tianjin Medical University Cancer Institute and Hospital between January 2009 and September 2015. All patients were firstly pathologically diagnosed with NSCLC and were classified according to the most recent International Association for the Study of Lung Cancer TNM classification system. All 106 enrolled patients had complete clinicopathological data, and all patients' postoperative follow-up information was documented by telephone (the median is 36 months, ranging from 12 to 90 months). Overall survival (OS) was defined as the period from the time of surgery to death or to the last follow-up. Disease-free survival (DFS) time was an interval between the time of surgery and the time when recurrence was diagnosed or the time of the last date of follow-up.

### 2.2. Immunohistochemical Staining and Analysis

The carcinoma and matched tissues were fixed in 10% formaldehyde, embedded in paraffin and cut into 4 *μ*m sections, and mounted on slides for immunohistochemical analysis. Immunohistochemistry was performed using the Histostain-Plus SP kit, which offers superior sensitivity. Briefly, the sections were baked at 70°C for 1 h and deparaffinised with xylene and rehydrated through gradient ethanol immersion. Endogenous peroxidase activity was quenched by 0.3% (*v*/*v*) hydrogen peroxide in methanol for 20 min and then washed three times with PBS. The sections were fixed with formaldehyde and blocked with 10% (*v*/*v*) normal goat serum in PBS for 1 h, followed by overnight incubation at 4°C with the anti-CIAPIN1 primary antibody diluted to 1 : 200 (initial concentration 1.8 mg/ml) in PBS containing 3% (*w*/*v*) BSA. After rinsing with PBS containing 0.02% (*v*/*v*) Tween-20 (PBST) three times 5 min each, sections were incubated with a biotinylated goat anti-mouse IgG polyclonal antibody for 20 min at 37°C. Then, the slides were rinsed and incubated with streptavidin-horseradish peroxidase for 20 min at room temperature followed by repeated washes, as described above. The reaction product was visualized with a 3,3′-diaminobenzidine (DAB) chromogen substrate at room temperature for 5 min. Sections were counterstained with haematoxylin for 30 sec and rinsed with tap water, were immediately dehydrated by sequential immersion in gradient ethanol and xylene, and were then mounted with Permount onto coverslips. Images were obtained under a light microscope equipped with a DP70 digital camera.

A negative control was performed by replacing the primary antibody with preimmune mouse serum. The sections were evaluated in consensus viewpoints by two pathologists who were blinded to the identity of the tissue. Any disagreement or uncertainty was resolved by consensus after joint review. To sum up, the IHC score was judged by the semiquantitative method that takes the synthesizing staining intensity and stained cell proportion together into account. Expression of CIAPIN1 was evaluated as the percentage of positive cells and staining intensity. (1) The percentage of positive cells was evaluated quantitatively and scored as “0” for staining of ≤1% of total cells counted, “1” for staining of 2~25%, “2” for staining of 26~50%, “3” for staining of 51~75%, and “4” for staining of >75% of the cells examined. (2) The intensity score was assigned for the average intensity of positive tumor cells (colorless: 0, pallid: 1, yellow: 2, and brown: 3). Overall, CIAPIN1 expression was graded as follows: low (-, +) and high (++, +++).

### 2.3. Cell Culture, Antibodies, and Reagents

The human NSCLC cell A549 was preserved by our laboratory. A549 cells were cultured in DMEM (HyClone, Logan, USA) containing 100 U/ml penicillin and 100 *μ*g/ml streptomycin (Gibco-BRL Life Technologies, Canada), supplemented with 10% FBS (HyClone, Logan, UT), at 37°C in a humid atmosphere with 5% CO_2_. When the growth of the cell monolayer reached 70%-80% confluence, 0.25% trypsin was used for digestion.

We purchased the NHE1-specific inhibitor Cariporide from Santa Cruz Biotechnology (Santa Cruz, CA, USA) and the MEK1 inhibitor PD98059 from Beyotime (Shanghai, China). For western blotting analysis, antibodies against CIAPIN1 (ab224172), MMP2 (ab97779), MMP9 (ab194316), and MMP14 (ab51074) were purchased from Abcam (MA, USA); antibodies against GAPDH (sc-47724), NHE1 (sc-136239), anti-p-ERK1/2 (sc-101760), and nonphosphorylated ERK1/2 (sc-81457) from Santa Cruz Biotechnology (Santa Cruz, CA, USA); and antibodies against snail (3895S), slug (9585S), twist (46702S), zeb1 (3396S), E-cadherin (14472S), cyclin D1 (2978S), cyclin D2 (3741S), cyclin D3 (2936S), cyclin E (4136S), cdk2 (2546S), cdk4 (12790S), Bax (14796S), Bid (8762S), Bim (2933S), Mcl-1 (94296S), and Bcl-2 (15071S) from Cell Signaling Technology (CST, MA, USA). The Human Phospho-MAPK Array Kit was purchased from R&D System (MN, USA). The ECL (enhanced chemiluminescence reagent plus) reagents were purchased from BD Transduction Laboratories (CA, USA).

### 2.4. The Virus Packaging and Generation of the Lentiviral Particles

The complete CDS of the CIAPIN1 gene was amplified from the template by PCR with the upstream primer 5′-gc TCTAGAATGGCAGATTTTGGG ATCTCTG-3′ and the downstream primer 5′-gc GAATTCCTAGGC ATCATGAAGATTGC-3′. The Xba I and Ecor I (Takara, Japan) enzyme sites were introduced in the primers. The multiple cloning site (MCS) of a pCDH-CMV-MCS-EF1-Green-Puro-CD513B-1 vector and the amplified CIAPIN1 PCR product were simultaneously digested by using Xba I and Ecor I (Takara, Japan) enzymes, and then the vector and PCR fragment were ligated by T4 DNA ligase during a 16 h reaction. The recombination vector was then transformed into the freshly prepared *E. coli* DH5*α* competent cells overnight at 37°C. The positive clones were picked up in LB agar plates covered with 100 *μ*g/ml ampicillin. Finally, the inserted CDS fragment in the pCDH-CMV-MCS-EF1-Green-Puro-CD513B-1 vector was sequenced for further identification, and the expression plasmid was named CIAPIN1-CD513B-1.

For each 10 cm dish, we prepared reagents as follows: solution A was prepared by mixing 10 *μ*g recombinant vector or scrambled control plasmid with 10 *μ*g Second-Generation Packaging Mix (pSPAX2 and pMD2.G) in 1 ml serum-free, antibiotic-free RPMI 1640 medium and letting it sit at room temperature for 5 min [20] and solution B was prepared by diluting 80 *μ*l Lentifectin™ transfection reagent in the same medium and letting it sit for 5 min [20]. Then, we mixed these two solutions and incubated it for 20 min at room temperature. After the 2 ml transfection complex was added to 1 × 10^7^ 293T cells in a logarithmic growth phase, 4.5 ml serum-free medium was transferred to the dish. After 293T cells were incubated at 37°C in a 5% CO_2_ atmosphere for 5-8 h, 0.5 ml FBS was added to the cultured cells (the method was described in the previous research by Zhang et al.) [21].

We evaluated the transfection efficiency 24 h posttransfection by observing the percentage of positive green fluorescent protein (GFP) cells under a fluorescence microscope. The transfection medium was removed and replaced with new 10% FBS RPMI 1640, and the cells were cultured for another 24-48 h. The culture supernatant was collected, filtered with a 0.45 *μ*m filter, and concentrated through ultracentrifugation. The viral particles were aliquoted and stored at -80°C.

### 2.5. The Establishment of the Transfected Human Lung Cancer Cell Line A549

Initially, the screening concentration of puromycin was determined as 400 ng/ml. Then, parental A549 cells were infected with the packaged virus particles and polybrene (8 *μ*g/ml) overnight, and the new culture medium containing 400 ng/ml puromycin was added the next day. The negative control (pCDH-CMV-MCS-EF1-Green-Puro-CD513B-1-nontarget vector) was simultaneously transfected into A549 cells. We changed 10% FBS medium with 400 ng/ml puromycin weekly until the drug-resistant clones were steadily obtained.

### 2.6. Real-Time Quantitative Reverse Transcription PCR and Western Blotting

The real-time quantitative reverse transcription PCR and the western blot technique were performed as described in our previously published research [22].

### 2.7. Wound Healing Assay

To examine and evaluate the migration ability of A549 cells *in vitro*, a wound healing (scratch) test was carried out. Cells of logarithmic phase were collected and then were cultured in 24-well plates until they reached 70% to 80% confluence. Afterwards, A549 cells were scratched by 20 *μ*l white pipette tips to construct an artificial wound gap. The migrating distance was measured at 24 h after scraping. The images have been taken by a camera (Model DXM1200, Nikon, Japan) installed in an inverted microscope (Eclipse TE300, Nikon, Japan).

### 2.8. Cell Invasion Assay

The Transwell chamber invasion assay was employed for cell invasion. Briefly, 8 *μ*M pore-covered polyvinylpyrrolidone-free polycarbonate membranes (Millipore, USA) were coated with BD Matrigel™ Matrix (15 *μ*g/filter), followed by incubation at 37°C for 1 h. Treated A549 cells were starved for 24 h, and then cells were trypsinized, centrifuged, and resuspended at a concentration of 10^7^ cells/ml in FBS-free DMEM. 5 × 10^5^ tumor cells in 200 *μ*l of serum-free media were added to the upper chamber, while the lower wells of the chamber contained 600 *μ*l DMEM medium with 10% FBS. After incubation for 24 h, the chamber was removed and the cells on Matrigel and the surface of the supper chamber were wiped off carefully with a cotton swab. Cells were fixed with 4% paraformaldehyde at room temperature for 10 min and stained with 0.5% crystal violet. The cells were counted and photographed with a light microscope at 200x. The central field and 6 peripheral fields were randomly selected to count the cells (the method was described in the previous research work by our research group) [23].

### 2.9. Tumorigenicity Assay

For *in vivo* analysis, we purchased BALB/c nude mice from the Shanghai Laboratory Animal Center of the Chinese Academy of Sciences. Mice were housed under specific pathogen-free conditions in the Experiment Animal Center of Tianjin Medical University Cancer Institute and Hospital. Mouse healthy status was monitored daily, and all protocols involved in the research were approved by the institutional biomedical research ethics committee of the Laboratory Animal Center of Tianjin Medical University Cancer Institute and Hospital. The A549 cells and A549 cells with CIAPIN1 overexpression in the exponential growth phase were harvested by brief exposure to a 0.25% trypsin/0.02% EDTA solution (*w*/*v*). Viable cells (2.0 × 10^6^ in PBS/100 *μ*l) were injected subcutaneously into the flanks of 4-week-old nude mice. The tumors were monitored with a caliper every week over a 6-week period after cell inoculation. The average tumor size was estimated by physical measurement of the excised tumor at the time of killing. Tumor volume for each mouse was determined (in cubic millimeter) by measuring in two directions and calculated as tumor volume = length × (width)^2^/2. Each experimental group consisted of five mice.

### 2.10. Statistical Analysis

Nominal variables were compared using the *χ*^2^ test, and ordinal categorical variables were evaluated by nonparametric Spearman's rank test. The Kaplan-Meier curve was done to calculate the patients' cumulative survival probabilities and compared using the log-rank test. The Cox proportional hazards model with likelihood ratio statistics was employed to further evaluate the risk factors for survival. All statistical analyses were conducted using SPSS version 13.0 software (SPSS, Chicago, IL). The significance of the *in vitro* data was determined with Student's *t*-test (two-tailed), whereas that of the *in vivo* data was determined with the two-tailed Mann-Whitney *U* test. In all of the tests, results were considered statistically significant for *P* < 0.05.

## 3. Results

### 3.1. CIAPIN1 Expression Was Decreased or Absent in NSCLC

**Figure 1:**
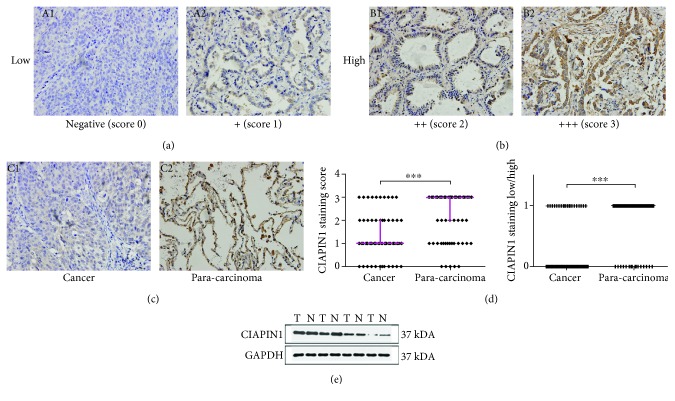
CIAPIN1 protein expression in NSCLC tissues and the matched noncancerous counterparts was detected. CIAPIN1 protein expressions were observed by immunohistochemical staining in 106 cases of NSCLC specimens. Representative photographs were taken at the magnification of 400x. (a) The immune scores 0 and 1 were defined as low CIAPIN1 expression. Representative photographs with low CIAPIN1 expression were shown. (b) The immune scores 2 and 3 were defined as high CIAPIN1 expression. Representative photographs with high CIAPIN1 expression were shown. Blue was marked as the nucleus, and brown was marked as the CIAPIN1 protein. (c) CIAPIN1 expression was relatively high in noncancerous tissues observed by immunohistochemical staining. (d) The CIAPIN1 staining score was statistically higher in matched noncancerous specimens compared with NSCLC samples (*P* < 0.001). (e) Western botting analysis was further performed to confirm the higher expression of CIAPIN1 in four matched benign tissues compared with cancer tissues. ^∗∗∗^*P* < 0.001, compared with control.

Here, we investigated the expression of CIAPIN1 in 106 NSCLC samples and 80 adjacent noncancerous counterparts. CIAPIN1 expression was classified as low (negative, +; score 0-1) and high (++, +++; score 2-3). The representative photographs were taken at a magnification of 400x (Figures 1(a) and 1(b)). By analysis of immunohistochemistry, it was found that CIAPIN1 was predominantly located in both the cytoplasm and nucleus of the adjacent benign tissue cells (Figure 1(c)). As shown in Table 1, the percentage of cells with high CIAPIN1 expression in NSCLC was 25% (20/80), significantly statistically lower than 80% (64/80) in the nontumor adjacent tissue (*P* < 0.001). The CIAPIN1 expression in NSCLC tumors was significantly lower than that in surrounding nontumor tissues. The average staining score in adjacent noncancerous tissue of NSCLC was significantly higher than that of NSCLC (*P* < 0.001) (Figure 1(d)). To further confirm these observations, the western blot assay was done using four paired tumor tissue specimens and matched normal tissues with known levels of CIAPIN1 expression by immunohistochemical staining. It was clear that the tumor tissue specimens had a decrease trend of CIAPIN1 expression as compared with the normal lung tissue, which was consistent with the level of CIAPIN1 protein expression determined by immunohistochemical staining (Figure 1(e)).

### 3.2. Correlation of CIAPIN1 Expression and Clinicopathological Characteristics

To further evaluate the correlation of CIAPIN1 expression levels and clinical parameters, the following study was done. As shown in Table 2, patients' characteristics such as age, gender, smoking status, histological type, TNM stage, tumor size, and lymphatic invasion were summarized. All the information was obtained from medical records. The diagnosis was confirmed by histological analysis by three pathologists. We found that the CIAPIN1 expression level was inversely correlated with histological type, TNM stage, tumor size, and depth of invasion (Table 2). By univariable and multivariate analysis, we found that tumor size, depth of invasion, and CIAPIN1 expression are the independent prognostic factors for OS, while tumor size and depth of invasion are the independent prognostic factors for DFS (Table 3). These data indicated that the expression level of CIAPIN1 is lower, the TNM stage is higher, the tumor size is larger, and the lymphatic invasion is deeper (*P* < 0.05). However, there was no correlation between CIAPIN1 expression and age, gender, and smoking status.

### 3.3. CIAPIN1 Depletion Is Associated with Poor Clinical Outcome of NSCLC Patients

**Figure 2:**
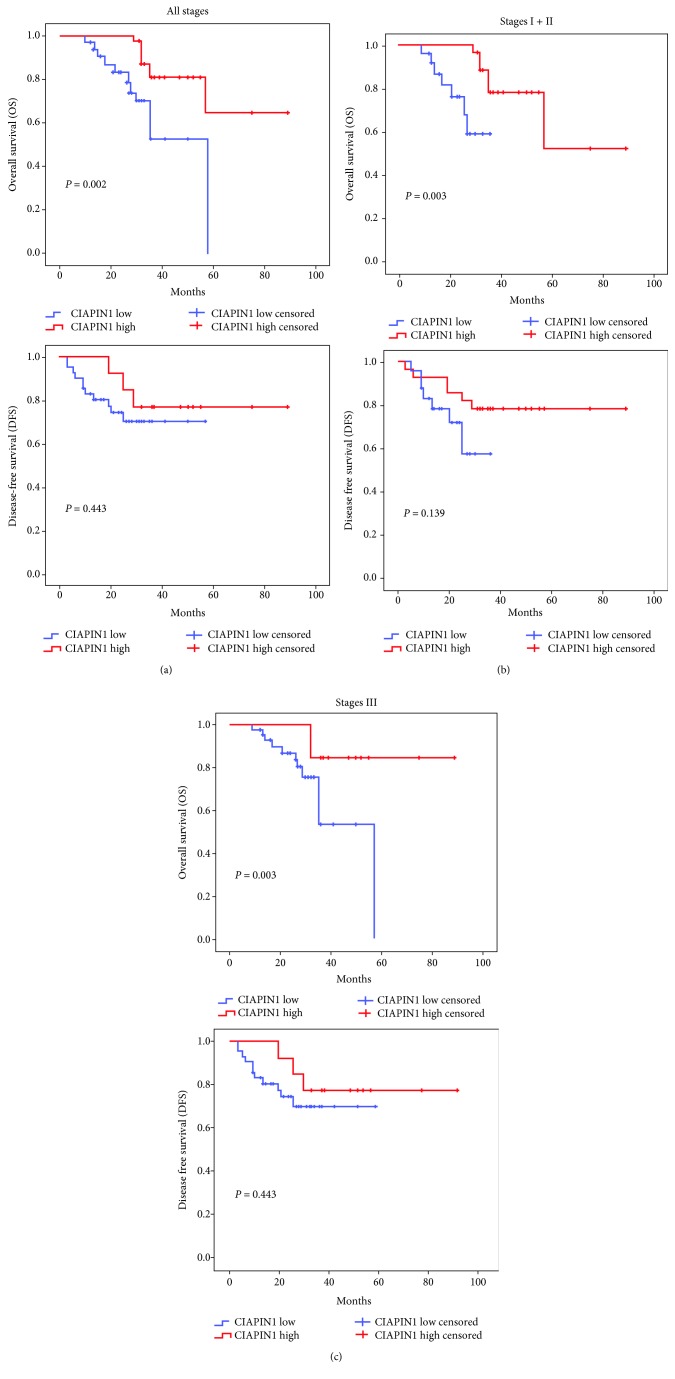
Good OS was correlated with CIAPIN1^high^ NSCLC cancer patients. Kaplan-Meier survival curves based on high vs. low expression levels of CIAPIN1 in NSCLC patients. (a) Patients in all stages. (b) Patients in stages I and II. (c) Patients in stage III.

The following study was performed to examine whether CIAPIN1 expression affects the cumulative survival probability. By adopting a log-rank test with Kaplan-Meier analysis, we found that different CIAPIN1 expressions could affect the overall survival (OS) and disease-free survival (DFS) (Table 3). According to follow-up results, the survival status of all-stage patients with variable CIAPIN1 expressions was further calculated. As shown in Figure 2, NSCLC patients with low CIAPIN1 expression revealed a relatively shorter OS duration when compared with patients with high CIAPIN1 expression. In the subsequent study, lung cancer patients were divided into early stage (stage I+stage II) and late stage (stage III), and then the survival status was estimated. The analysis results showed that the CIAPIN1 expression was higher and the prognosis was better in both early stage and late stage. All the above data implied that CIAPIN1 might be considered a valuable prognostic marker for the survival of NSCLC patients. Thus, we hypothesize that CIAPIN1 might exert inhibitory function in tumor biological behavior such as metastasis of NSCLC cells. To elucidate our hypothesis, the subsequent experiments were carried out.

### 3.4. Overexpression of CIAPIN1 Inhibited A549 Cells' Migration and Invasion

**Figure 3:**
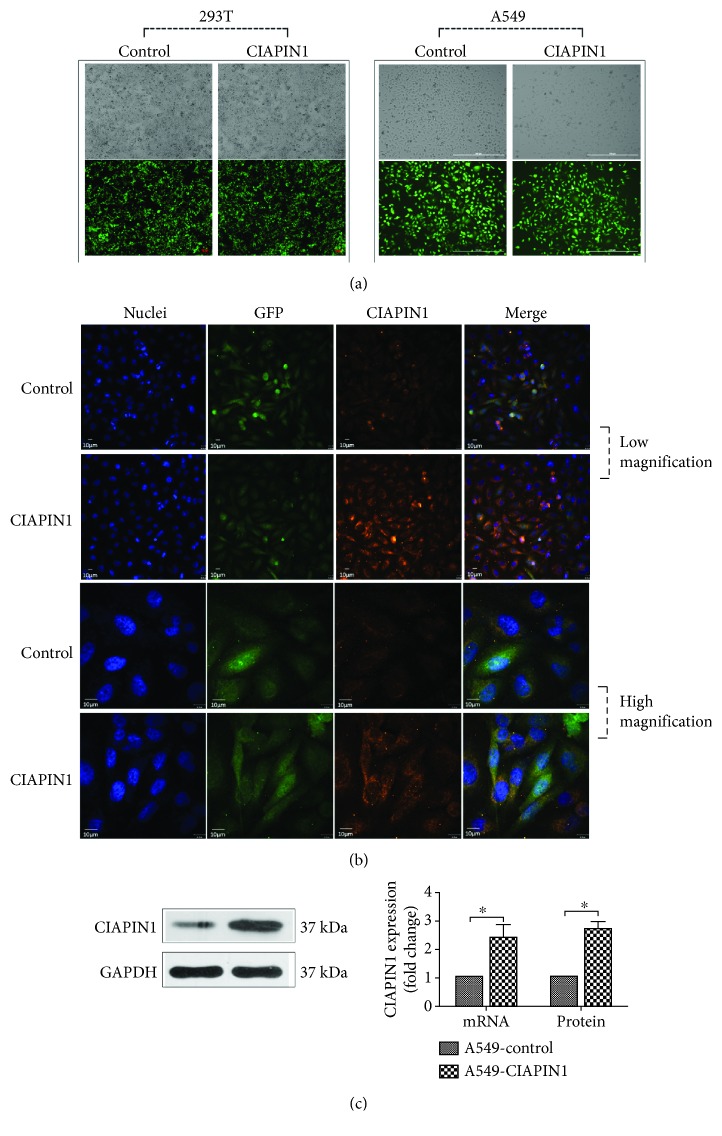
Validation of CIAPIN1 overexpression was assessed in A549 cells. (a) The representative photographs of 293T cells and A549 cells with GFP expression were shown. (b) The immunofluorescence assay was performed to detect the increase in CIAPIN1 observed with a confocal laser microscope. Blue indicated nuclei, green indicated GFP, and red indicated CIAPIN1 staining. (c) The mRNA and protein expressions of CIAPIN1 in A549 cells with CIAPIN1 overexpression were detected by real-time quantitative PCR and western blotting. Each value indicated the mean ± SD of three independent experiments. ^∗^*P* < 0.05, compared with control.

In the following study, we successfully altered CIAPIN1 expression in A549 cells to explore the role of CIAPIN1 in lung cancer cells' migration and invasion. The lentiviral plasmid with CIAPIN1 inserted and the control vector were packaged in 293T cells. The representative photographs of 293T cells which expressed GFP were shown (Figure 3(a)). Then, the CIAPIN1 plasmid and the control vector were transfected into A549 cells, and the representative photographs of A549 cells which expressed GFP were shown (Figure 3(a)). Immunofluorescence staining was also performed to confirm the high expression of CIAPIN1 with a confocal laser microscope (Figure 3(b)). After observation of GFP expressions in A549 cells, the real-time quantitative PCR and western blotting assays were conducted to test the overexpression efficacy of CIAPIN1. The measurement showed that the CIAPIN1 expression was upregulated at both mRNA and protein levels (Figure 3(c)).

**Figure 4:**
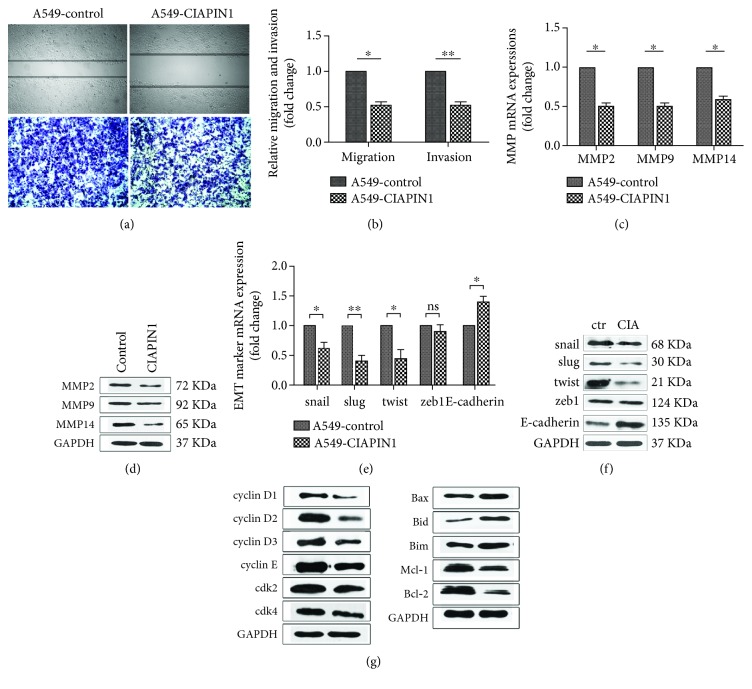
Effect of CIAPIN1 upregulation on NSCLC A549 cells' metastatic potential was measured. (a) Representative images of cells' migration and invasion were taken. (b) The relative cells' migration and invasion after CIAPIN1 upregulation were measured. (c, d) The mRNA expression and the protein levels of MMP2, MMP9, and MMP14 were detected by real-time quantitative PCR and western blotting. (e, f) The mRNA expression and the protein levels of snail, slug, twist, zeb1, and E-cadherin were detected by real-time quantitative PCR and western blotting. (g) Expressions of cell cycle- and apoptosis-associated proteins were detected by western blotting. The expressions of cyclin D1, cyclin D2, cyclin D3, cyclin E, cdk2, and cdk4 were detected in A549 cells with CIAPIN1 overexpression. The expressions of Bax, Bid, Bim, Bcl-2, and Mcl-1 were detected in A549 cells with CIAPIN1 overexpression. GAPDH was used as an internal control. Each value indicated the mean ± SD of three independent experiments. ^∗^*P* < 0.05, ^∗∗^*P* < 0.01, compared with control.

A549 cells are characterized by their high metastatic potential. The results showed that A549 cells' metastatic potential was suppressed by upregulation of CIAPIN1 via the wound healing and the Transwell assays (Figure 4(a)). Our data demonstrated that both the amount of A549 cells that migrated to the lower side of the membrane and the wound closure speed of A549 cells were slackened obviously along with CIAPIN1 overexpression (*P* < 0.05, Figure 4(b)). Tumor metastasis is mainly mediated by degradation of ECM. MMPs have shown pivotal roles in this process. We then perform western blotting to detect the expressions of MMP2, MMP9, and MMP14 in A549 cells with CIAPIN1 overexpression. Both the mRNA and protein expression levels of all these three MMPs were decreased when compared with A549 control cells (*P* < 0.05, Figures 4(c) and 4(d)). In addition, epithelial-to-mesenchymal transition (EMT) is critical for tumor metastasis [24, 25] and some EMT-specific markers were detected in A549 cells with CIAPIN1 overexpression. We found that snail, slug, and twist were decreased and E-cadherin was increased along with CIAPIN1 upregulation. No difference was found in zeb1 expression (*P* < 0.05, Figures 4(e) and 4(f)).

It is known that the classic cell cycle regulator cyclins and cyclin-dependent kinase (cdk) contributed to cell cycle initiation and progression. Beyond the detection of metastasis-related MMP proteins, we also investigated whether the cell proliferation status was affected. We detected cell cycle- and apoptosis-associated proteins with CIAPIN1 upregulation. The current data clearly demonstrated that CIAPIN1 was correlated with the reduction of cyclin D1, cyclin D2, cyclin D3, cyclin E, cdk2, cdk4, Mcl-1, and Bcl-2, as well as with the increase in Bax, Bid, and Bim (Figure 4(g)). These data suggested that CIAPIN1 upregulation could inhibit cells' metastatic potential and proliferation via regulating MMPs, EMT, and specific cell cycle effectors.

### 3.5. NHE1 and ERK1/2 Might Contribute to the Inhibition of A549 Cells' Metastatic Potential Conferred by CIAPIN1 Upregulation

**Figure 5:**
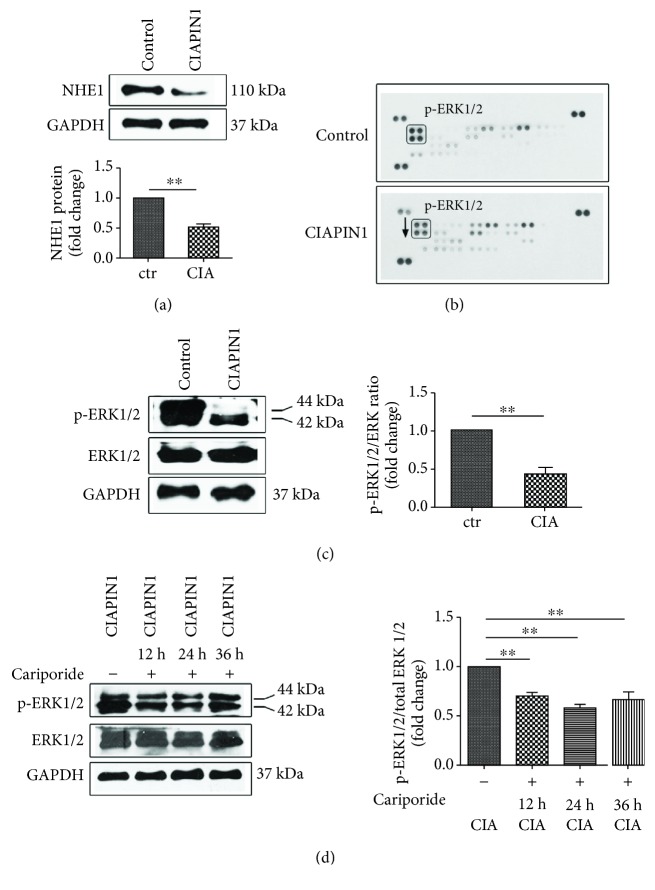
NHE1 and ERK1/2 signaling pathway might contribute to cells' metastatic potential conferred by CIAPIN1 upregulation. (a) The NHE1 protein expression was decreased in A549 cells with CIAPIN1 upregulation. (b, c) The protein array and western blotting were conducted to detect the ERK1/2 signaling pathway changes in A549 cells with CIAPIN1 upregulation. (d) ERK1/2 phosphorylation was further modulated by the NHE1 inhibitor at the indicated time in A549 cells with CIAPIN1 overexpression. Each value indicated the mean ± SD of three independent experiments. ^∗∗^*P* < 0.01, compared with control.

We found the decreased expression of NHE1 in A549 cells with CIAPIN1 overexpression compared with A549 control cells (*P* < 0.01, Figure 5(a)). We further performed the protein array assay to detect the MAPK protein changes, and the results demonstrated that p-ERK1/2 was blocked (Figure 5(b)). Western blotting was further employed to confirm the decreased p-ERK1/2 (*P* < 0.01, Figure 5(c)). We also found that phosphorylated ERK1/2 was regulated by NHE1 activity in A549 cells in CIAPIN1 overexpression. The activity of ERK1/2 in A549 cells in CIAPIN1 overexpression was detected after Cariporide treatment. The western blotting results demonstrated that NHE1 inhibition decreased ERK1/2 phosphorylation in A549 cells with CIAPIN1 overexpression at 12 h and 24 h (*P* < 0.01, Figure 5(d)). Collectively, these results indicated that the NHE1 and ERK1/2 signaling pathway might play pivotal roles in CIAPIN1 upregulation conferred by a decrease in metastatic potential of A549 cells.

### 3.6. Blockage of the NHE1 and ERK1/2 Signaling Pathway Contributed to the Inhibition of A549 Cells' Metastasis

**Figure 6:**
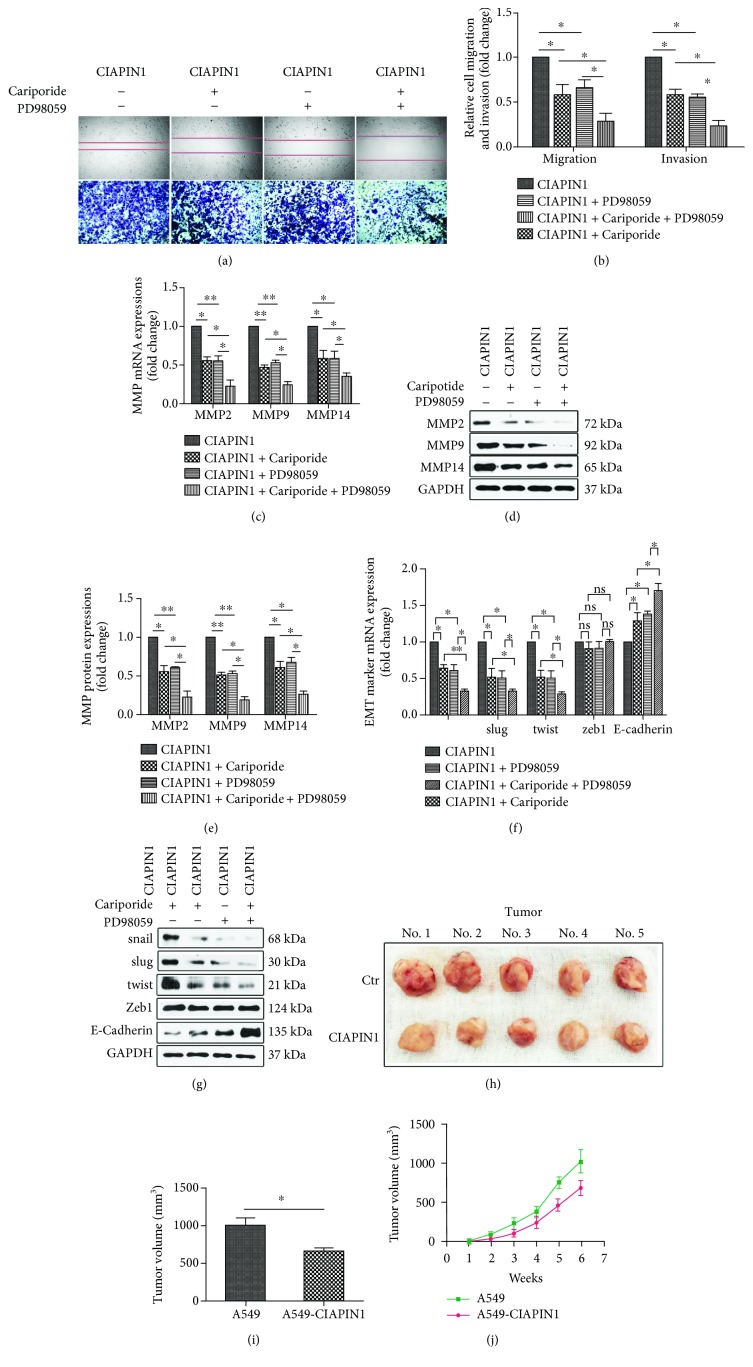
ERK1/2 signaling pathway was involved in A549 cells' metastasis conferred by CIAPIN1. (a) A549 cells with CIAPIN1 overexpression were treated with Cariporide or PD98059, and then cells' migration and invasion were measured by wound healing and Transwell assays. (b) Changes of A549 cells' migration and invasion after indicated treatments were detected. (c–e) Cariporide or PD98059 was added to A549 cells with CIAPIN1 overexpression, and the expressions of MMP2, MMP9, and MMP14 were detected. The quantification of MMPs was shown. (f, g) Cariporide or PD98059 was added to A549 cells with CIAPIN1 overexpression, and the expressions of snail, slug, twist, zeb1, and E-cadherin were detected. (h, i) CIAPIN1 upregulation led to the suppression of tumor volume. Tumor volume was calculated for a period of 6 weeks. (j) CIAPIN1 upregulation led to decreased tumor outgrowth analyzed by the tumor growth curve. Each value indicated the mean ± SD of three independent experiments. ^∗^*P* < 0.05, ^∗∗^*P* < 0.01, compared with control.

We then utilized a specific MEK1 pharmacological inhibitor, PD98059, to block the ERK1/2 signaling pathway, and then the metastatic potential was detected. As shown in Figures 6(a) and 6(b), the migration and invasion of A549 cells with CIAPIN1 overexpression were further suppressed by the use of either Cariporide or PD98059. Treatment with either Cariporide or PD98059 induced almost similar blockage of metastasis in A549 cells with CIAPIN1 overexpression. Further, we proved that Cariporide and PD98059 exerted a synergistic effect in suppressing migration and invasion of A549 cells with CIAPIN1 upregulation (*P* < 0.05, Figures 6(a) and 6(b)). Moreover, as expected, treating A549 cells with CIAPIN1 overexpression with Cariporide or PD98059 resulted in nearly the same inhibition of MMP and EMT marker expressions (Figures 6(c)–6(g)). Importantly, these two specific inhibitors employed together could induce the maximum inhibition of MMPs and EMT markers (*P* < 0.05, Figures 6(c)–6(g)). These data implied that the ERK1/2 signaling pathway had an essential role in regulating the metastatic potential of A549 cells under CIAPIN1 overexpression condition.

### 3.7. Upregulation of CIAPIN1 Suppressed Lung Tumor Growth

To test the hypothesis that CIAPIN1 can negatively regulate the tumor growth, the parental A549 cells and A549 cells with CIAPIN1 upregulation were injected subcutaneously into the flanks of 4-week-old nude mice. Of note, A549 cells engineered to overexpress CIAPIN1 showed a marked decrease in the onset of the first palpable tumor. Moreover, A549 cells with CIAPIN1 overexpression showed a significant decrease in tumor outgrowth after injection when compared with control cells. The representative photographs of tumors were shown (Figure 6(h)). The mean tumor size of scramble cells at 36 days was 1017 mm^3^, whereas the mean tumor sizes of A549 cells with CIAPIN1 overexpression were 667 mm^3^ (Figure 6(i)). The tumor growth curve was shown (Figure 6(j)).

## 4. Discussion

CIAPIN1 was a recently identified apoptosis inhibitor whose biological function was far from being thoroughly understood. Hao et al. detected the CIAPIN1 distribution in a series of cultured cell lines, such as mouse fibroblast cell line NIH3T3, human tumor cell lines, and immortalized cell lines by the use of molecular techniques of immunofluorescence staining, immunohistochemistry, and EGFP-CIAPIN1 fusion protein [12]. The intracellular distribution of CIAPIN1 has been demonstrated to play a cell death-defying role in a variety of mouse and human embryonic cells [11, 12, 16, 26]. Furthermore, CIAPIN1 expression was observed in organs of a 5-month-old human embryo, and the expression level of CIAPIN1 in the 5-month-old human embryo was higher than that in adult organs of endodermal and ectodermal origin. But the result in the organs of mesoderm origin was opposite [16]. All these previous findings suggested that CIAPIN1 was also expressed in normal tissues and was closely associated with differentiation.

Some previous studies confirmed that liver cancer, gastric cancer, and clear cell renal cell carcinoma (CCRCC) revealed relatively high CIAPIN1 expression compared to their matched adjacent tissue counterparts, while interference of CIAPIN1 might participate in carcinogenesis of colorectal cancer and HCC cells reflecting on proliferation and cell cycle promotion both *in vitro* and *in vivo* [17, 27–29]. The report indicated that CIAPIN1 might be considered a candidate tumor suppressor gene in CCRCC, while effects of CIAPIN1 on lung cancer remain unknown. Initially, we investigated the expression of CIAPIN1 in lung cancer with the purpose of seeking the working molecular mechanisms of NSCLC metastatic potential and ultimately finding a novel molecular marker for early diagnosis and prognosis of NSCLC patients. Our results revealed high CIAPIN1 expression in lung cancer, and CIAPIN1 was closely associated with the prognosis of NSCLC patients. The CIAPIN1 expression was higher, and the prognosis of NSCLC patients was better. Lower CIAPIN1 expression predicted a worse outcome for NSCLC patients. CIAPIN1 could be considered a potentially significant prognostic indicator for NSCLC patients, which might be useful for clinicians to provide individualized therapy for NSCLC patients with optimal benefit, while a study by other research groups demonstrated that CIAPIN1 expression has a prognostic value in several human tumors [26, 29, 30] and revealed that patients with high levels of CAIPIN1 nuclear expression displayed shorter postoperative survival times than those with weak nuclear expression. The distinguishable role of CIAPIN1 might be due to the tumor types, indicating that CIAPIN1 exerted variable roles in different cancers.

After demonstrating the prognosis value of CIAPIN1 in NSCLC patients, we then investigated its role in A549 cells and found that CIAPIN1 upregulation inhibited cells' metastatic potential both *in vivo* and *in vitro*. Tumor cells need to overcome a series of multiple biological obstacles to form a metastatic tumor. It is known that matrix metalloproteinases (MMPs) contribute to tumor metastasis [31]. MMPs are a family of zinc-dependent matrix-degrading proteases that have a crucial role in tumor metastasis, in which MMP2, MMP9, and MMP14 are the three major proteinases among MMP subfamily members involved in pericellular proteolysis associated with cell migration [32, 33]. Our current study found that CIAPIN1 upregulation reduced the expressions of MMP2, MMP9, and MMP14. In addition, epithelial-to-mesenchymal transition (EMT) is critical for tumor metastasis and some EMT symbol markers were detected in A549 cells with CIAPIN1 overexpression. We found that snail, slug, and twist decreased and E-cadherin was increased along with CIAPIN1 upregulation.

To gain insights into how CIAPIN1 mediated metastatic capacity of A549 cells, we then investigated which signaling pathways participated in this process. Tumor invasion and metastasis are multistage and multifactorial processes, which are regulated by complicated mechanisms and multiple signaling pathways. In recent years, an increasing body of evidence shows that MAPKs could activate and phosphorylate the substrates to regulate the proliferation, differentiation, apoptosis, and metastasis of cells [34]. In our present study, we investigated the role of the MAPK signaling pathway in A549 cells' migration and invasion. Our results showed that CIAPIN1 upregulation suppressed ERK1/2 phosphorylation. We then speculated that blockage of the ERK1/2 signaling pathway might contribute to the suppression of A549 cells' metastatic capacity conferred by CIAPIN1 overexpression. To further explore the effect of blocking the ERK1/2 signaling pathway to CIAPIN1-mediated metastatic capacity, we used the ERK1/2-specific inhibitor PD98059 and measured A549 cells' migration and invasion. We found that ERK1/2 inhibition could further suppress the metastatic capacity in A549 cells in CIAPIN1 upregulation.

The Na+/H+ exchanger isoform 1 (NHE1) is almost quiescent in normal cells, but in tumor cells, the hyperactivated NHE1 results in an increase in pH and acidification of the extracellular space. However, little is known about the signal transduction system regulation by NHE1 activity, and that is associated with tumor cell invasiveness [35]. Recent studies have demonstrated that NHE1 activity and expression increased in a variety of tumor cells [36]. Our results showed that NHE1 decreased along with CIAPIN1 upregulation, while whether NHE1 inhibition contributed to A549 cells' metastasis suppression needs to be elucidated. We then treated A549 cells with CIAPIN1 upregulation with the NHE1 inhibitor Cariporide and measured the metastatic potential. As our expectation, Cariporide targeting NHE1 significantly further suppressed the metastatic capacity of A549 cells with CIAPIN1 upregulation.

A growing body of literature implicates that NHE1 regulates the activities of ERK1/2, JNK, and p38 MAPK, yet the effects of NHE1 on MAPK activity are highly context- and cell type-specific [37]. For example, Pedersen et al. reported that NHE1 inhibited ERK1/2 but activated JNK and p38 MAPK after osmotic shrinkage in Ehrlich Lettre Ascites cells [38]. In our study, we found that inhibition of NHE1 could reduce the expression of phosphorylated ERK1/2 in A549 cells with CIAPIN1 upregulation. To further investigate whether the metastatic suppression exerted by CIAPIN1 upregulation depended on the NHE1 and ERK1/2 signaling pathway, the NHE1 inhibitor Cariporide and the ERK1/2 inhibitor PD98059 were applied. As shown in our results, supplementation with Cariporide or PD98059 resulted in nearly the same reduction of CIAPIN1-mediated metastatic capacity. All these results indicated the regulation axis CIAPIN1-NHE1-ERK1/2 in A549 cells. All these findings indicated that CIAPIN1 targeted NHE1 and ERK1/2 to suppress the metastatic capacity of A549 cells.

To sum up, our results revealed that CIAPIN1 expression differed in cancer tissues and their matched adjacent tissues and high CIAPIN1 expression predicted good prognosis for NSCLC patients. To our knowledge, this is the first to associate CIAPIN1 expression with NSCLC patients' prognosis. Our present findings suggest that CIAPIN1 may not only be a useful indicator for predicting the outcome of lung cancer patients but also be an effective therapeutic target for lung cancer. All the work provided a potential management by which CIAPIN1 might be crucial on the suppression of lung cancer metastasis. The current work implied that CIAPIN1 combined with anticancer chemotherapeutical agents would be a potential effective strategy. To better understand the exact molecular mechanism for the aberrant CIAPIN1 signaling pathway, further detailed research is required, which may help us to design an effective therapeutic modality to control lung cancer.

## Figures and Tables

**Table 1:** CIAPIN1 expression in NSCLC specimens and paired normal tissues.

Samples	Total number of cases	CIAPIN1 expressions	
		Low	High
NSCLC	80	60 (75%)	20 (25%)
Adjacent nontumorous specimens	80	16 (20%)	64 (80%)

**Table 2:** Correlation between CIAPIN1 expression levels and clinicopathological parameters (*P* < 0.05 statistically significant).

Parameters	Total (*N* = 106)	CIAPIN1 expressions			
		Low	High	Chi-square(*χ*^2^)	*P*
Age (year)				1.057	0.304
<60	48 (45%)	32	16		
≥60	58 (55%)	33	25		
Gender				1.143	0.285
Male	63 (59%)	36	27		
Female	43 (41%)	29	14		
Smoking status				0.012	0.913
Never smoker	51 (48%)	31	20		
Smoker	55 (52%)	34	21		
Histological type				8.585	0.003 (*P* < 0.05)
Squamous	67 (63%)	34	33		
Adenocarcinoma	39 (37%)	31	8		
TNM stage				12.569	0.001 (*P* < 0.05)
I and II	52 (49%)	23	29		
III	54 (51%)	42	12		
Tumor size				14.116	0.001 (*P* < 0.05)
≤3	67 (63%)	32	35		
>3	39 (37%)	33	6		
Lymphatic invasion				22.092	0.001 (*P* < 0.05)
Negative	38 (36%)	12	26		
Positive	68 (64%)	53	15		

**Table 3:** Univariate analysis of the association between clinical characteristics and survival in patients with NSCLC (*P* < 0.05 is considered statistically significant).

Variables	Univariate analysis		Multivariate analysis	
	Chi-square	*P*	HR (95% CI)	*P*
*OS*				
Age (<60 years vs. ≥60 years)	0.171	0.679		
Gender (male vs. female)	0.090	0.765		
Smoking status (never smoker vs. smoker)	2.760	0.097		
Histological type (squamous cell carcinoma vs. adenocarcinoma)	2.819	0.093		
TNM stage (I, II, and III)	4.236	0.040	0.644 (0.198-2.100)	0.465
Tumor size (≤3 cm vs. >3 cm)	6.874	0.009	0.397 (0.164-0.961)	0.040
Lymphatic invasion (negative vs. positive)	11.712	0.001	0.247 (0.086-0.704)	0.009
CIAPIN1 expression (low vs. high)	7.401	0.007	3.120 (1.084-8.977)	0.035
*DFS*				
Age (<60 years vs. ≥60 years)	0.003	0.959		
Gender (male vs. female)	0.106	0.745		
Smoking status (never smoker vs. smoker)	2.113	0.146		
Histological type (squamous cell carcinoma vs. adenocarcinoma)	1.668	0.197		
TNM stage (I, II, and III)	3.089	0.079		
Tumor size (≤3 cm vs. >3 cm)	7.961	0.005	0.354 (0.145-0.865)	0.023
Lymphatic invasion (negative vs. positive)	8.603	0.003	0.309 (0.115-0.828)	0.020
CIAPIN1 expression (low vs. high)	4.677	0.031	2.120 (0.784-5.734)	0.139

## Data Availability

All data generated or analyzed during this study are included in this published article.
